# Targeting the nuclear factor kappa B pathway for the medical management of intervertebral disc degeneration

**DOI:** 10.3389/fnins.2026.1874921

**Published:** 2026-08-03

**Authors:** Jiansen Wang, Kaixu Han, Dongfeng Li, Yukang Qin, Jing Zhang, Zhinan Ren, Lei Yu, Guangduo Zhu, Cheng Peng, Yingjie Hao

**Affiliations:** Department of Orthopedics, The First Affiliated Hospital of Zhengzhou University, Zhengzhou, Henan, China

**Keywords:** IVDD, medical management, natural compounds, NF-κB pathway, novel materials

## Abstract

Intervertebral disc degeneration (IVDD), a key contributor to low back pain, is driven by multiple predisposing factors, such as oxidative stress, inflammation, and mechanical stress, among others, severely impacting patients’ quality of life. The nuclear factor kappa B (NF-κB) signaling pathway is crucial in the development of IVDD. This review examines the mechanisms of NF-κB activation, focusing on classical and non-classical pathways and their roles in IVDD progression. A variety of therapeutic approaches have been developed to target the NF-κB pathway, including NSAIDs, proteins, non-coding RNAs, hormones, natural chemicals, and sophisticated biomaterials. These interventions inhibit NF-κB activation, alleviating apoptosis, extracellular matrix (ECM) imbalance, and inflammation in the nucleus pulposus. In this review, we not only focus on summarizing the emerging biomaterials and natural medicines targeting NF-κB to treat IVDD in recent years, but also discuss the current challenges, such as limited drug delivery to the avascular disc and the need to elucidate non-classical NF-κB pathways, and outline future directions for clinical translation. These insights inject new vitality into the treatment of IVDD. Despite progress in targeting NF-κB, significant challenges remain in translating these therapies into clinical practice. Further exploring the potential of targeting the NF-κB pathway in the treatment of IVDD can promote the development of new IVDD treatments.

## 1 Introduction

Low back pain (LBP) is a major global clinical problem that profoundly impairs patients’ quality of life and imposes a heavy socioeconomic burden. A principal pathological contributor to LBP is intervertebral disc degeneration (IVDD), a common degenerative disorder of the spine. Globally, the incidence of IVDD is approximately 10%, and its prevalence rises markedly with age (Jin Y. et al., 2026). Although IVDD predominantly affects individuals over 40, it can also occur in younger populations, often driven by unhealthy lifestyle habits. LBP represents the cardinal symptom of IVDD and the primary reason patients seek medical care (Mei et al., 2026).

The intervertebral disc is composed of the annulus fibrosus (AF), nucleus pulposus (NP), and cartilage endplate (CEP) and functions to connect adjacent vertebrae while maintaining spinal stability (Zeng et al., 2023). Loss of nucleus pulposus cells (NPCs) and disruption of extracellular matrix (ECM) homeostasis represent key pathological features of IVDD, both of which are closely associated with activation of the nuclear factor kappa B (NF-κB) pathway in NPCs (Li et al., 2026; Yao et al., 2026). This pathway can be triggered by a range of pathological stimuli, including oxidative stress, inflammation, nutrient deprivation, and excessive mechanical loading (Sun K. et al., 2024). Once activated, NF-κB directly promotes ECM degradation by upregulating matrix metalloproteinases (MMPs) and a disintegrin and metalloproteinase with thrombospondin motifs (ADAMTS) and simultaneously induces NPC apoptosis and senescence, thereby serving as a central driver of IVDD (Nian et al., 2026). In parallel, NF-κB stimulates the release of pro-inflammatory cytokines such as interleukin-1β (IL-1β) and tumor necrosis factor-α (TNF-α) and amplifies oxidative stress through a positive feedback loop, further aggravating structural and cellular damage within the disc (Wang et al., 2026). Accordingly, the NF-κB pathway is increasingly recognized as a key signaling hub that integrates upstream injurious signals and orchestrates downstream destructive cascades in IVDD.

This review offers an in-depth account of the distinct role of the NF-κB pathway in IVDD and highlights recent advances in the identification of inhibitory targets within this pathway. A defining feature of the article is its dedicated emphasis on two emerging directions, namely plant-derived natural compounds and novel biomaterials, systematically examining their capacity to modulate NF-κB signaling. By bringing these innovative areas into focus, we aim to provide a fresh perspective on therapeutic strategies for IVDD.

## 2 The structure of intervertebral discs

**Figure 1:**
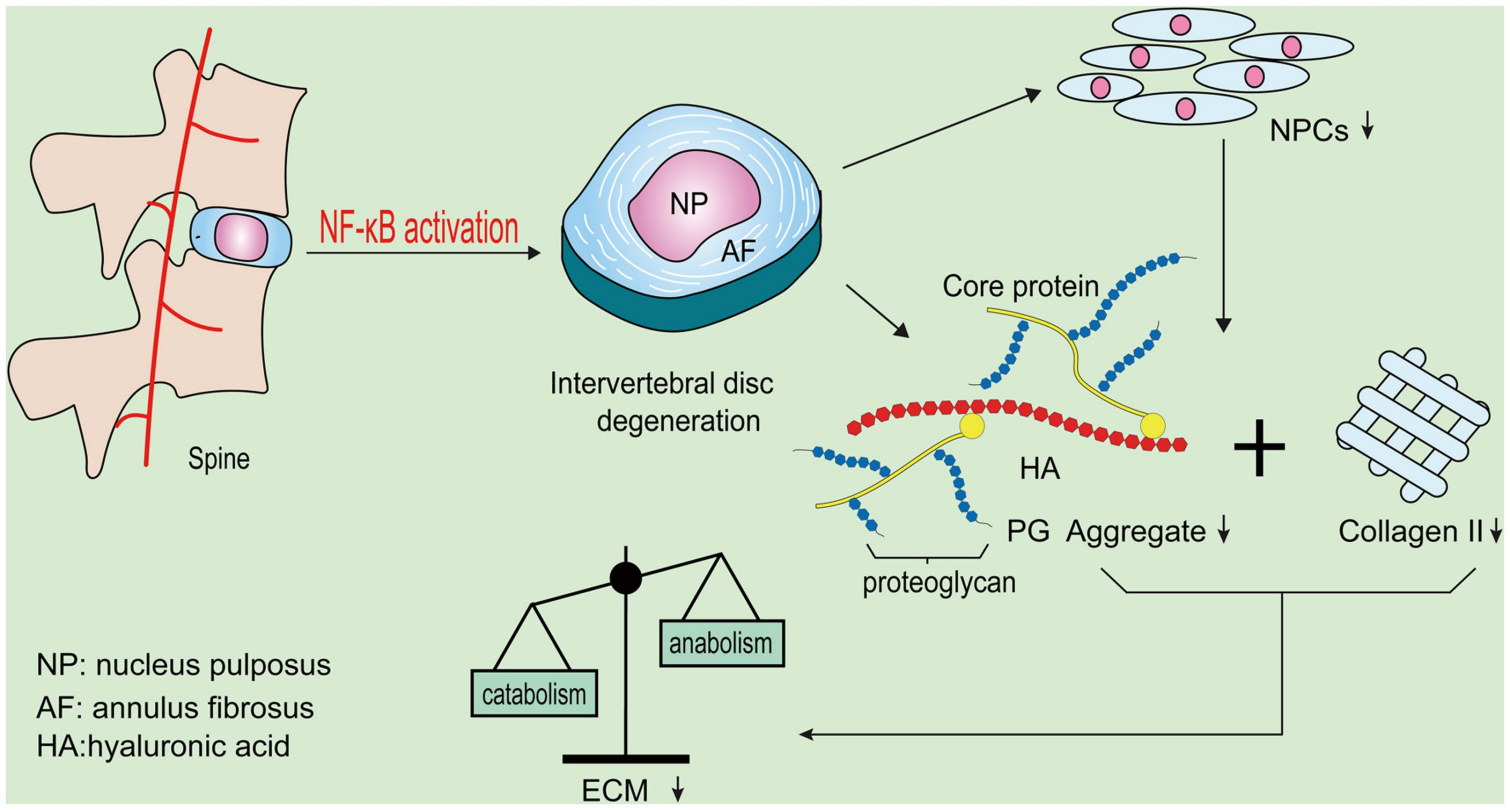
Damage to intervertebral disc structures caused by IVDD.

The human spine contains 23 intervertebral discs, primarily composed of the NP, AF, and CEPs. The NP is located at the center of the intervertebral disc and is a gel-like tissue with a high water retention capacity (Pu et al., 2023). NPCs synthesize the extracellular matrix (ECM), an amorphous matrix primarily composed of proteoglycan (PG) aggregates, collagen, elastic fibers, and lipids (Sun K. et al., 2024; Wei et al., 2020). PG aggregates are relatively complex, primarily consisting of a non-covalent combination of PGs and hyaluronic acid (HA) (Ming Long and Zong Ping, 2004). PGs consist of one or more glycosaminoglycan (GAG) chains attached to a core protein, with HA being a type of GAG (Xinzheng et al., 2023). In PG aggregates, HA forms the central filament, and one end of the core protein of multiple PGs is non-covalently bound to it. PG aggregates and collagen in the NP contribute significantly to the water retention capacity of the intervertebral disc. This water retention function helps to distribute pressure laterally from the spine to the surrounding tissues, significantly enhancing the elasticity and support of the intervertebral disc. PG aggregates contain numerous GAG chains, which carry a high negative charge, enabling them to attract and bind water molecules (Priscilla et al., 2014). The collagen in the NP is predominantly type II, containing multiple hydroxylysine residues, with its imino acid side chains glycated to form disaccharide and monosaccharide derivatives (Marta et al., 2023). These structures can accumulate a significant number of water molecules, maintaining the NP in a highly hydrated state (Figure 1).

AF is a fibrous ring around the NP, composed of inner and outer layers rich in type II and type I collagen. From the inner to the outer layer, the type I/type II ratio increases, while glycosaminoglycan and water contents decline (Zhang A. et al., 2023). The AF restricts NP motion, transmits compressive loads, and limits excessive spinal movement (Molladavoodi et al., 2020). In IVDD, the lamellar architecture breaks down, giving rise to centrifugal cracks. CEPs supply nutrients to the intervertebral disc via capillaries from the vertebral arteries (Bonnheim et al., 2022). With aging and IVDD, these endplates undergo progressive thinning and calcification and compromise disc nutrition.

## 3 Overview of the NF-κB pathway

The human body relies on NF-κB, a family of transcription factors, for several physiological and pathological functions. And there is a DNA-interacting Rel homology domain (RHD) at the N-terminus of every member of the family (Deng et al., 2020; Matthew and Sankar, 2014). Additionally, distinct protein domains regulate transcription by recruiting coactivators and corepressors, thereby exerting both positive and negative effects. NF-κB typically functions as a dimer, composed of proteins from the Rel family.

There are two pathways of NF-κB activation. Both the classical and non-canonical pathways include NF-κB dimers, with the former including p50 and p65 proteins and the latter including p52 and RelB. These two pathways are activated and regulated by distinct mechanisms, leading to different impacts on IVDD (Sun, 2017).

### 3.1 Activation and regulation of the classical NF-κB pathway

During activation of the NF-κB classical pathway, the NF-κB transcription factor exists as a protein dimer composed of p50 and p65. Under normal conditions, p50 and p65 dimers are inhibited by the inhibitor of NF-κB (IκB), causing p50, p65, and IκB to form a complex in the cytoplasm, preventing their entry into the nucleus to initiate gene transcription (Smaldone et al., 2021). The IκB kinase (IKK) complex, located upstream of the NF-κB transcription factor, can relieve the inhibition caused by IκB proteins and promote nuclear transcription. The catalytic subunits IKKα (IKK1) and IKKβ (IKK2), as well as the regulatory component NF-κB essential modulator (NEMO), make up IKK (Simonsen et al., 2021; Jin W. et al., 2026).

**Figure 2:**
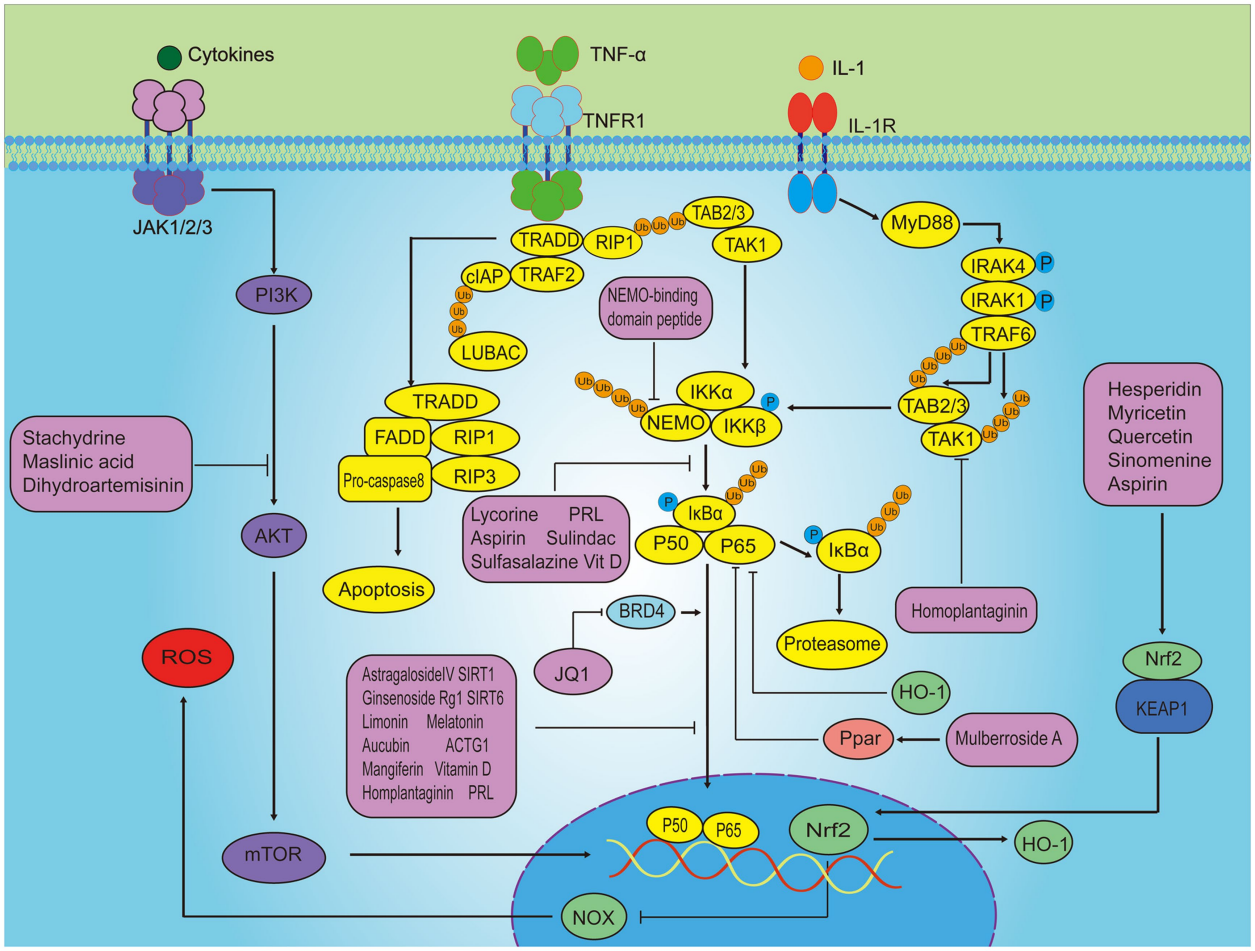
NF-κB classical activation pathway and the targets of various substances.

Among these, IKKβ activates the classical NF-κB pathway, while IKKα mediates the non-canonical NF-κB pathway. NEMO does not have catalytic activity but is essential for the activation of the classical NF-κB pathway (Pu et al., 2023; Lin et al., 2023). The N-terminal region of NEMO connects IKKα and IKKβ to maintain the integrity of IKK, while its C-terminal region receives and transmits signals upstream of the pathway (Glaeser et al., 2020). Unphosphorylated IκB and p50-p65 dimers are closely linked, inhibiting their nuclear translocation and subsequent functionality. IKKβ phosphorylates IκB, targeting it for ubiquitination and subsequent proteasomal degradation. This degradation disrupts the interaction between IκB and the p50-p65 dimers, thereby releasing the dimers to translocate into the nucleus and activate gene transcription (Pourdad and Pourdad, 2026) (Figure 2).

Given the significant upregulation of TNF-α and IL-1β in IVDD and their role as potent activators of the classical NF-κB pathway, it is necessary to focus on elucidating their signaling mechanisms. The vast majority of predisposing factors for IVDD, such as mechanical overload, oxidative stress, microenvironmental acidification, and aging, can directly or indirectly promote the release of these two pro-inflammatory cytokines, making them a common downstream hub for various pathological stimuli. Once these factors activate the NF-κB pathway, they in turn enhance their own transcription, forming a positive feedback loop that ultimately accelerates the degenerative process. Therefore, a detailed analysis of the classical NF-κB activation pathway in NPCs is highly representative.

#### 3.1.1 TNF-α

After TNF-α activates TNFR1, TNFR1 recruits TNF receptor-associated death domain protein (TRADD). TRADD acts as an adaptor and recruits receptor-interacting protein 1(RIP1) and TRAF2 through its TRAF-binding domain (RIP1 also contributes to TRAF2 recruitment). The TRAF2 trimer recruits cellular inhibitor of apoptosis protein 1/2 (cIAP1/2), which further recruits linear ubiquitin chain assembly complex (LUBAC). The recruitment of the IKK complex is achieved through multiple mechanisms: (1) TRAF2 directly binds IKKα/*β*; (2) RIP1 recruits IKK and transforming growth factor-β-activated kinase 1 (TAK1) complexes via NEMO; (3) TAK1 is closely associated with the recruitment and activation of IKK, promoting IKK phosphorylation and activation. During this process, cIAP1/2 and LUBAC participate in the activation of TAK1 and IKK by generating linear or K63-linked ubiquitination modifications (Wang et al., 2020).

TNF-α drives IVDD via a different repertoire of mediators. It upregulates prolyl hydroxylase 2 and 3 (PHD2/3) to synergistically enhance the transcription of ADAMTS-5 and MMP-13. It specifically induces the chemokines CCL20, CXCL2 and CXCL5, as well as the adhesion molecule ICAM-1, which promote neutrophil infiltration. TNF-α also elevates cyclin B1 and Notch1/2 to stimulate pathological hyperproliferation, activates caspase-8 to initiate extrinsic apoptosis, and uniquely induces the autophagy-related gene p62, implicating autophagic stress in the degenerative process.

#### 3.1.2 IL-1β

IL-1β first binds to IL-1RI, inducing a conformational change and recruiting IL-1 receptor accessory protein (IL-1RAcP) via the extracellular domain, forming a high-affinity ternary complex. This complex promotes dimerization of the intracellular Toll/IL-1 receptor (TIR) domains, which in turn recruits the adaptor protein myeloid differentiation primary response 88 (MyD88). MyD88 recruits IL-1R-associated kinase 4 (IRAK4) and IRAK1, and IRAK4 phosphorylates and activates IRAK1, which then recruits TNF receptor-associated factor 6 (TRAF6). TRAF6 activates the TAK1-TAB complex, and the downstream signaling cascade is consistent with the TNFR1 pathway (activation of IKK phosphorylation, degradation of IκB, release of NF-κB) (Wang et al., 2020).

IL-1β promotes IVDD through a distinct set of downstream effector molecules. It primarily induces syndecan-4 (SDC4) to potentiate aggrecan degradation mediated by ADAMTS-5. It also strongly upregulates vascular endothelial growth factor (VEGF), brain-derived neurotrophic factor (BDNF) and nerve growth factor (NGF), thereby facilitating aberrant angiogenesis and sensory innervation. Concurrently, IL-1β elevates the senescence markers p16 and p53 and increases the pro-apoptotic protein Bax, thus accelerating both cellular senescence and apoptosis of NPCs.

### 3.2 Activation and regulation of the non-canonical NF-κB pathway

During the activation of the non-canonical NF-κB pathway, NF-κB is present as a protein dimer comprising p52 and RelB. In contrast to the classical pathway, p52, and RelB protein dimers lack IκB inhibitory factors (Sun, 2017).

P100, the precursor of p52, also functions as an IκB-like molecule that predominantly retains RelB in the cytoplasm, thereby inhibiting its nuclear translocation. The cIAP1/2-TRAF2-TRAF3 E3 ubiquitin ligase complex targets freshly synthesized NF-κB-inducing kinase (NIK) for ubiquitination-dependent destruction under steady-state conditions, preventing its accumulation. However, when a specific ligand binds to the receptor, TRAF3 is degraded, which leads to the accumulation of NIK (Deng et al., 2020). NIK then phosphorylates and activates IKKα, allowing NIK and IKKα to cooperatively mediate the phosphorylation and ubiquitin-dependent processing of p100 to generate p52.

**Figure 3:**
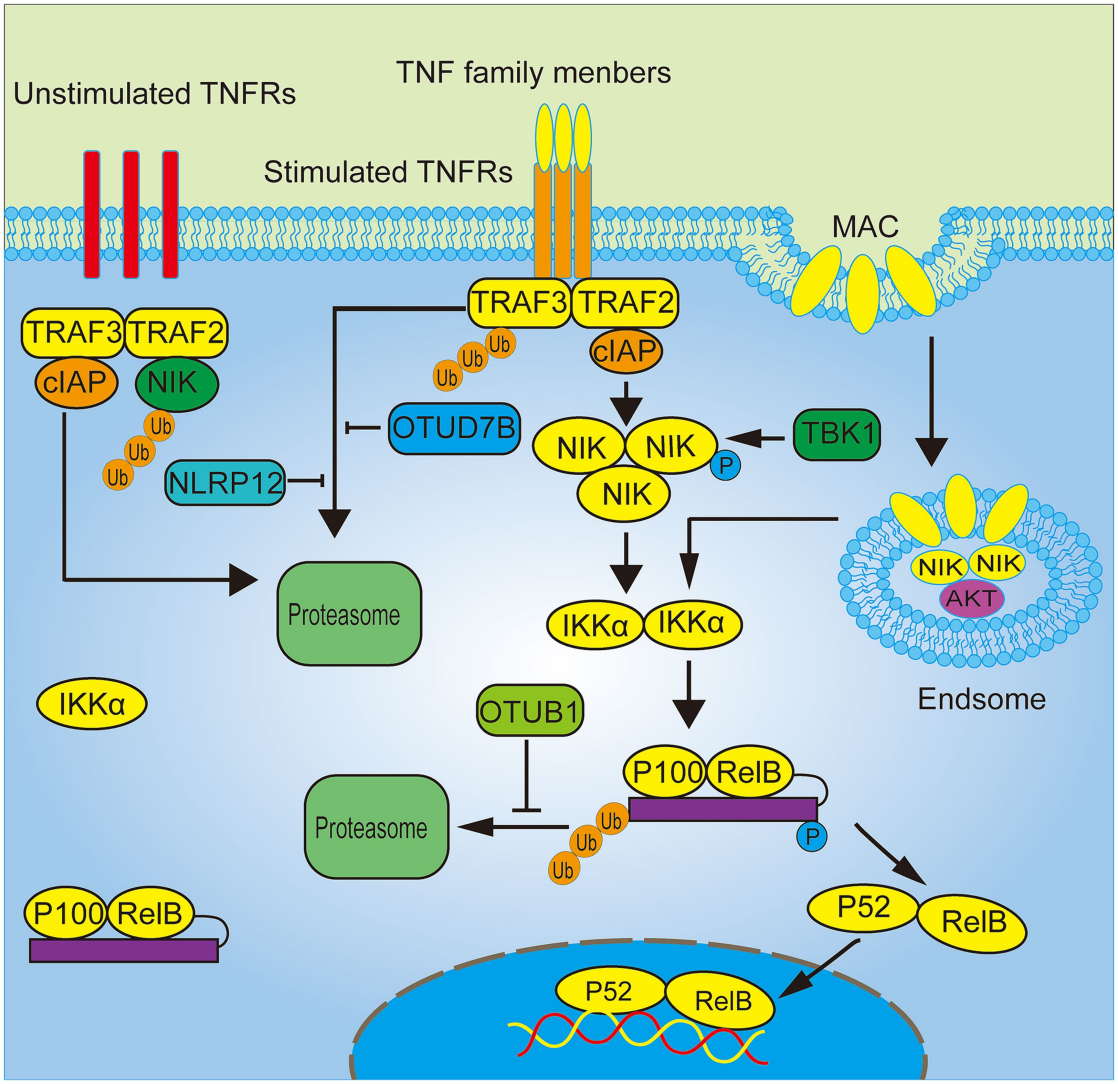
NF-κB non-canonical activation pathway.

The processing of p100 is a critical step in triggering the NF-κB non-canonical pathway (Dash et al., 2020). Besides being the precursor of p52, p100 also acts as an IκB-like molecule, primarily obstructing RelB nuclear translocation. Consequently, the RelB/p52 heterodimer is able to enter the nucleus and complete gene transcription once the restriction of p100 is relieved (Yu H. et al., 2020; Li X. et al., 2023; Sun, 2011) (Figure 3).

## 4 NF-κB pathway and IVDD

**Figure 4:**
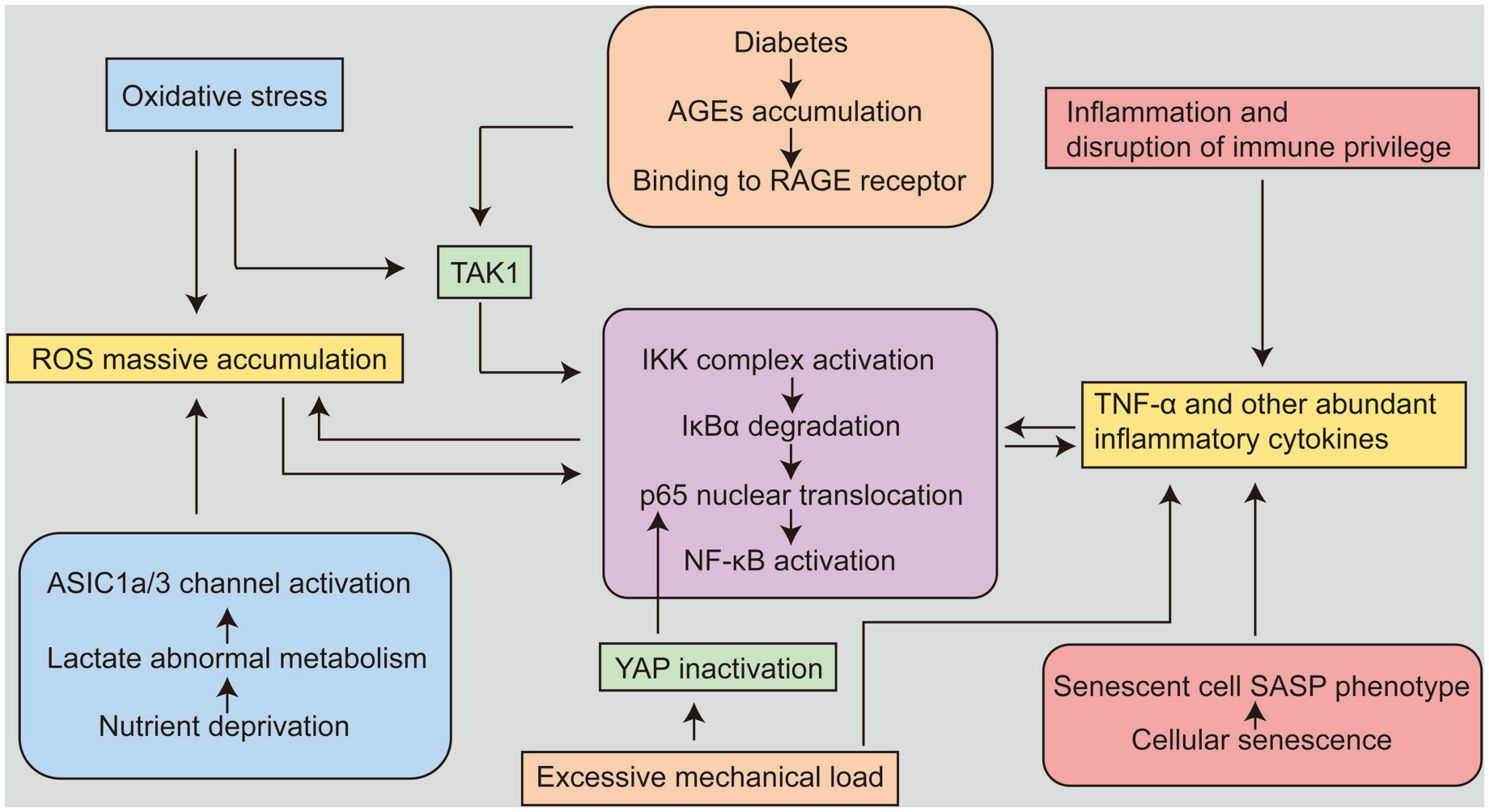
Mechanisms of NF-κB pathway activation by pathogenic factors in intervertebral disc degeneration.

Multiple pathogenic factors contribute to IVDD, including oxidative stress, excessive mechanical loading, pro-inflammatory stimuli, and nutrient deprivation, all of which drive IVDD progression through various signaling pathways. Among these pathways, the NF-κB signaling pathway is particularly significant. Therefore, this review focuses on how various pathogenic factors regulate the NF-κB pathway and influence IVDD, a crucial step in developing therapeutic strategies targeting the NF-κB pathway (Figure 4).

### 4.1 Oxidative stress

Oxidative stress is a recognized contributor to IVDD, closely linked to the activation of the NF-κB signaling pathway. The excessive generation of reactive oxygen species (ROS) and compromised antioxidant defense intensify the harm inflicted by oxidative stress (Li et al., 2021; Dang et al., 2020). The buildup of ROS initiates a series of cellular processes, notably the activation of NF-κB.

ROS activate the NF-κB pathway primarily through two distinct mechanisms. The first involves direct oxidative modification of the IKK complex: ROS oxidize the Cys179 residue within the catalytic domain of IKKβ to generate sulfenic acid (–SOH) or an intramolecular disulfide bond, which induces IKKβ autophosphorylation and subsequent activation of the IKK complex (Reynaert et al., 2006). The second mechanism engages crosstalk between the mitogen-activated protein kinase (MAPK) and NF-κB signaling cascades. ROS stimulate upstream MAP3Ks of the MAPK pathway, such as ASK1 and TAK1. Upon activation, these MAP3Ks act as IKK kinases, directly phosphorylating IKKβ to trigger activation of the IKK complex (Pantano et al., 2006). Activation of the NF-κB signaling pathway greatly upregulates the level of inflammatory factors and matrix metalloproteinases (MMPs), resulting in the destruction of the ECM in the intervertebral disc (Yang et al., 2021; Che et al., 2020). Elmounedi et al. (2024) developed a rat IVDD model and observed increased oxidative stress markers in IVDD. Furthermore, ozone treatment effectively inhibited oxidative stress and blocked multiple signaling pathways, including NF-κB, thereby alleviating IVDD symptoms.

### 4.2 Mechanical load

The influence of mechanical loading on IVDD is multifaceted. Previous studies have demonstrated that appropriate mechanical loading may have therapeutic effects on IVDD, while excessive loading exacerbates the condition (Wu et al., 2022; Wang H. et al., 2023; Chang et al., 2019; Wang S. et al., 2018). Researchers have reached varied conclusions regarding the mechanism by which excessive mechanical stress influences the NF-κB pathway. The NF-κB pathway is activated when chondrocytes are subjected to severe mechanical stress, according to research on osteoarthritis by Chang et al. (2019). Wang H. et al. (2023) discovered that moderate mechanical stimulation mitigates inflammation in annulus fibrosus cells by inhibiting NF-κB signaling via YAP. Excessive mechanical loading reduces Yes-associated protein (YAP) expression, resulting in increased nuclear translocation of p65 and activation of the NF-κB pathway. Huang et al. (2024) suggested that high-amplitude, low-frequency cyclic mechanical strain promotes degeneration of human NPCs and increases inflammatory cytokine secretion. When the NF-κB signaling pathway is activated, IVDD is worsened because NPCs upregulate IL-1β and other pro-inflammatory molecules in response to excessive mechanical stress. Although there is some inconsistency in the results, these studies do point to the fact that mechanical stress affects IVDD via controlling the NF-κB pathway. The variability in findings may stem from differences in the species of NPCs used in the experiments or from the interplay of multiple mechanisms. The regulation of the NF-κB signaling pathway results from the integrated action of various signaling pathways.

### 4.3 Inflammation and disruption of immune privilege

The formation of an inflammatory environment is a critical aspect of IVDD progression. Levels of various inflammatory factors, including TNF-α, IL-1α/*β*, interleukin-6 (IL-6) and interleukin-17 (IL-17), are significantly elevated in IVDD, and degenerated NPCs release substantial amounts of chemokines. These chemokines activate inflammatory cells such as macrophages, neutrophils, T cells, and B cells, thereby exacerbating the inflammatory environment (Huang et al., 2024; Sun et al., 2022). The inflammatory factors within this environment promote IVDD through several distinct signaling pathways. For instance, IL-1β, which activates the NF-κB pathway, can form a positive feedback loop with this pathway. In degenerated NPCs, IL-1β activates the NF-κB signaling pathway, which, in turn, upregulates IL-1β expression. The NF-κB pathway interacts with IL-1β, further exacerbating the severity of IVDD (Kagoya et al., 2014).

Beyond the direct actions of these inflammatory mediators, the structural disruption that occurs during degeneration progressively dismantles the immune privilege of the intervertebral disc. Under healthy conditions, the disc is an immune-privileged site owing to its avascular nature and the presence of immunoregulatory molecules such as Fas ligand. During degeneration, however, this structural disruption exposes normally sequestered disc-specific antigens to the systemic immune system, thereby promoting immune cell infiltration and eliciting adaptive immune responses (Sun et al., 2020). NF-κB activation within both disc cells and infiltrating immune cells drives macrophage polarization toward a pro-inflammatory M1 phenotype and activates T-cell responses. This establishes a self-perpetuating cycle of inflammation and matrix destruction that further aggravates IVDD.

### 4.4 Nutrient deprivation

Nutrient deprivation can significantly impair disc health, as intervertebral disc tissue has a limited blood supply and depends on diffusion for nutrient delivery. In healthy intervertebral discs, the NP, CEPs, and AF are nourished by a network of capillaries; however, blood vessels must traverse the CEPs. During degeneration, the CEPs undergo calcification, leading to an insufficient nutrient supply to the intervertebral disc. Under nutrient-deprived conditions, lactate accumulates abundantly within the intervertebral disc. The accumulated lactate stimulates acid-sensing ion channels (ASIC1a/3), leading to calcium influx and a burst of ROS. In turn, ROS activate NF-κB, which primes the NOD-like receptor family pyrin domain containing 3 (NLRP3) inflammasome and promotes pyroptosis (Zhao K. et al., 2021). Enhancing nutrient supply to the intervertebral disc could prevent or reverse IVDD. Understanding the interplay between nutritional status and activation of NF-κB may guide the formulation of innovative treatment approaches to preserve disc health.

### 4.5 Additional factors that engage NF-κB in IVDD

In addition to the four established triggers, multiple pathological factors have been demonstrated to directly or indirectly activate the NF-κB pathway in the intervertebral disc, including diabetes and cellular senescence. Under diabetic conditions, the accumulation of advanced glycation end products (AGEs) and their subsequent binding to the receptor for AGEs (RAGE) induce K63-linked polyubiquitination of TRAF6, which in turn activates TAK1 and consequently triggers the NF-κB signaling pathway (Liu et al., 2023). In degenerated discs, senescent cells adopt a senescence-associated secretory phenotype (SASP) and release substantial amounts of TNF-α and IL-1β. These cytokines not only activate NF-κB directly but also establish a positive feedback loop with the p53/p16 senescence pathways, thereby amplifying inflammatory and catabolic responses (Song et al., 2024). Collectively, these findings demonstrate that diverse pathogenic stimuli converge on the NF-κB pathway to drive IVDD, underscoring that therapeutic targeting of this central hub may provide a broad range of strategies for IVDD treatment.

### 4.6 Signaling crosstalk in IVDD: NF-κB convergence with MAPK and PI3K/Akt pathways

Most pathogenic drivers of IVDD do not act through a single pathway; instead, they concurrently modulate the NF-κB, MAPK, and phosphoinositide 3-kinase/protein kinase B (PI3K/Akt) signaling pathways. Extensive crosstalk exists among these pathways, centered on two key aspects. First, ROS and TAK1 serve as shared upstream regulators that simultaneously activate the NF-κB and MAPK pathways (Wei et al., 2026; Zhang X. et al., 2023). Within the pathological microenvironment of IVDD, excessive ROS accumulation activates TAK1 through oxidative modification. Activated TAK1 then phosphorylates both the IKK complex and MAPK kinases (MKKs), initiating the NF-κB and JNK/p38 MAPK cascades, respectively. This synchronized activation amplifies inflammatory signals and accelerates extracellular matrix degradation. Second, in contrast to the cooperative pro-degenerative actions of NF-κB and MAPK, the PI3K/Akt pathway negatively regulates NF-κB signaling (Ouyang et al., 2017). Activated Akt directly phosphorylates and stabilizes IκBα, thereby preventing NF-κB nuclear translocation; Akt can also phosphorylate and inhibit TAK1 kinase activity, thus dampening the upstream activation signals for NF-κB. Dissecting this crosstalk network helps identify critical interventional nodes and provides a foundation for combined targeted therapies against IVDD.

## 5 Treatments based on NF-κB pathway

### 5.1 NSAIDs

Nonsteroidal anti-inflammatory drugs (NSAIDs) are commonly used to alleviate low back pain. While their classical mechanism involves cyclooxygenase inhibition and prostaglandin synthesis blockade, certain NSAIDs (aspirin, sulindac, and sulfasalazine) also exert anti-inflammatory effects by impeding NF-κB activation.

Aspirin mitigates IVDD progression through multiple mechanisms. It reduces oxidative stress by lowering ROS and upregulating nuclear factor erythroid 2-related factor 2 (Nrf2) (Liu et al., 2019). A functional interplay exists between Nrf2 and NF-κB: decreased Nrf2 enhances NF-κB activity and cytokine output, while NF-κB reciprocally modulates Nrf2 transcription (Yang S. et al., 2024). Aspirin also suppresses pro-inflammatory cytokines and reduces ECM-degrading enzymes, preserving ECM homeostasis in NPCs (Kim et al., 2017). Mechanistically, aspirin activates AMP-activated protein kinase (AMPK) in a dose-dependent manner to reduce oxidative stress (Ren et al., 2022), and uniquely among NSAIDs, it competitively inhibits the IKKβ ATP-binding site, blocking IκB phosphorylation and p65 nuclear translocation (Wang Y. F. et al., 2023).

Sulindac and sulfasalazine also inhibit the NF-κB pathway: sulindac binds to IKKβ’s active site, while sulfasalazine suppresses both IKK*α* and IKKβ activities to prevent TNF-α- and LPS-induced NF-κB activation. Collectively, these NSAIDs offer therapeutic benefits for IVDD.

### 5.2 Growth factor-based therapies

Growth factors (GFs) are a class of proteins or polypeptides typically secreted by cells. They influence cell function by binding to specific surface receptors, activating intracellular signaling pathways, and regulating gene expression. These factors are crucial during embryonic development, tissue regeneration, and repair. Based on their structure and the receptor families they activate, GFs can be classified into four categories: (1) Class I GFs principally encompass transforming growth factor (TGF) and epidermal growth factor (EGF) (Noch et al., 2021; Abdul Basit et al., 2022). They are essential for cell growth and differentiation. (2) Class II GFs primarily include fibroblast growth factors (FGFs), which are key in embryonic development, wound healing, and angiogenesis (Chiara et al., 2015). (3) Class III GFs include platelet-derived growth factor (PDGF), which primarily promotes angiogenesis and cell migration (Mouton, 2023). (4) Class IV GFs are typically associated with nerve growth factor (NGF) and neurotrophic factors (Bartesaghi et al., 2022). They primarily function in the nervous system, promoting the growth and survival of nerve cells.

Relief and treatment of IVDD can be achieved by injecting GFs into the intervertebral discs to promote ECM production, prevent degeneration, and reduce inflammation (Walsh et al., 2004; Saarang et al., 2021). Regarding the NF-κB signaling pathway, GFs can modulate NF-κB through crosstalk with other signaling pathways, including MAPK and PI3K/Akt. In IVDD mouse models, the blockade of PDGF-BB receptors accelerates ECM degradation. More recent studies have shown that PDGF-BB significantly reduces IL-1*β* and IL-18 production, boosts the proliferation of NPCs, and increases the levels of aggrecan and COL2 (Weikang et al., 2022). In addition to these effects, GDF-5 (a member of the TGF-β superfamily) inhibits the calcification of CEPs and AFs, while facilitating nutrient transport to the intervertebral disc via the AFs, thereby preventing IVDD (Sheng et al., 2021). However, research by Walsh et al. (2004) suggests that not all GFs are involved in IVDD. GFs such as GDF-5 and PDGF-BB exhibit more pronounced therapeutic effects on IVDD, whereas insulin-like growth factor-1 and FGFs are less effective.

### 5.3 Protein and peptide-based therapies

The NF-κB signaling pathway is a complex and intricate physiological and biochemical process. The activation of this pathway involves multiple proteins and enzymes and is regulated by various other proteins. Certain proteins inhibit NF-κB activation, such as sirtuins, actin gamma 1 (ACTG1), and NEMO-binding domain peptides. Activating these proteins, it may aid in the treatment of IVDD. Moreover, certain proteins or peptides, like BRD4 and CGRP (neuropeptide), can initiate the NF-κB signaling pathway. The strategic use of inhibitors targeting these proteins is an effective approach to treating IVDD.

#### 5.3.1 Proteins that inhibit the NF-κB pathway

Sirtuins are evolutionarily conserved NAD^+^-dependent deacetylases that modify both histone and non-histone proteins. Mammals have seven sirtuins (SIRT1–SIRT7), each with distinct roles and subcellular distributions. SIRT1 and SIRT6 inhibit the NF-κB pathway, offering therapeutic potential for IVDD.

Sirtuin 1 (SIRT1) deacetylates the NF-κB p65 subunit, promoting its nuclear-to-cytoplasmic translocation and suppressing its transcriptional activity. Downregulation of SIRT1 increases p65 and p-p65 levels, reducing type II collagen and aggrecan while elevating TNF-α, IL-1β, and IL-6 (Sun H. J. et al., 2021). Resveratrol and vitamin D activate SIRT1, which deacetylates p65 and blocks its nuclear translocation, thereby promoting NPC proliferation, inhibiting senescence, and preserving ECM synthesis (Wang P. et al., 2023; Yang et al., 2019; Ciccone et al., 2022). Thus, SIRT1 activation may be particularly beneficial for IVDD associated with vitamin D deficiency, and the 1,25 (OH)₂D/SIRT1 axis represents a promising therapeutic target (Zhou et al., 2022; Sabir et al., 2017).

Sirtuin 6 (SIRT6) serves as a potent antagonist in IVDD, with its expression downregulated in degenerative NP tissues and inversely correlated with disease severity. Mechanistically, SIRT6 is recruited to the promoters of NF-κB target genes, where it deacetylates H3 lysine 9 (H3K9) to repress transcription. Although SIRT6 overexpression does not affect IL-1β-induced p65 nuclear translocation or IκBα degradation, it directly suppresses p65 transcriptional activity, thereby mitigating extracellular matrix degradation (Kang et al., 2017).

ACTG1 also modulates IVDD via NF-κB. ACTG1 levels are lower in severely degenerated NP than in mild NP, and ACTG1 knockdown upregulates p-p65, indicating its protective role (Wang et al., 2024). Similarly, LIF delays IVDD by inhibiting p65 phosphorylation/nuclear translocation (Wu et al., 2021). The NEMO-binding domain (NBD) peptide interferes with NF-κB activation; its local injection reduces IL-6 and alleviates disc dehydration and degeneration (Glaeser et al., 2020). These findings support NBD peptides as potential future therapeutics for IVDD.

#### 5.3.2 Proteins that activate the NF-κB pathway

Bromodomain-containing protein 4 (BRD4), a member of the BET family, activates the NF-κB pathway and is linked to chronic inflammation. Its inhibitor JQ1 has been studied for antitumor efficacy (Park et al., 2019). In IVDD, BRD4 levels correlate with MMP-13, whose expression is regulated via MAPK and NF-κB in NPCs. BRD4 inhibition (via lentiviral knockdown or JQ1) suppresses these pathways, reducing p65 phosphorylation and increasing IκBα, thereby lowering MMP-13 and alleviating IVDD (Wang et al., 2019).

Calcitonin gene-related peptide (CGRP) is a 37-amino acid neuropeptide. Its expression and receptor levels are elevated in degenerated discs, directly promoting IVDD. CGRP inhibits NPC proliferation, induces apoptosis, disrupts ECM homeostasis, and activates inflammatory responses through the MAPK and NF-κB pathways (Afroz et al., 2020). Rimegepant, a CGRP receptor antagonist, ameliorates CGRP-mediated IVDD *in vitro* (Sun K. et al., 2021). The CGRP-Calcrl/RAMP1 axis drives IVDD via NF-κB/MAPK, positioning CGRP receptors as potential therapeutic targets.

### 5.4 Non-coding RNA

Non-coding RNAs (ncRNAs) are RNA transcripts that are transcribed from the genome and are not translated into proteins. They are classified into circular RNAs (circRNAs) and linear ncRNAs; linear ncRNAs include long non-coding RNAs (lncRNAs) and small non-coding RNAs (sncRNAs), such as microRNAs (miRNAs), siRNAs, and piRNAs (Singh and Roy, 2022; Yu et al., 2025; Ke et al., 2025). MiRNAs directly modulate signaling pathways or protein activity, whereas lncRNAs and circRNAs often act as miRNA sponges. In IVDD, ncRNAs are dysregulated—Lan et al. (2016) found 50 of 2059 miRNAs altered in degenerated discs. Several ncRNAs modulate the NF-κB pathway, thereby influencing IVDD progression and representing promising therapeutic targets (Table 1).

**Table 1:** Effects of non-coding RNAs on the NF-κB signaling pathway.

Non-coding RNAs	Target gene(s)	Role in IVDD	Mechanism of action	References
miRNAs				
miR-141	SIRT1	Promote degeneration	Downregulate SIRT1 → Reduce deacetylation of p65 at Lys310 → Enhance p65 transcriptional activity and NF-κB activation	Ji et al. (2018)
miR-217	SIRT1	Alleviate degeneration	Upregulate SIRT1 → Promote deacetylation of p65 → Suppress p65 transcriptional activity and NF-κB activation	Tao et al. (2024)
miR146a	TRAF6	Alleviate degeneration	Downregulate mRNA and protein levels of TRAF6 → Reduce TRAF6-mediated activation of TAK1/IKK complex → Inhibit IκBα phosphorylation and p65 nuclear translocation → Suppress NF-κB signaling	Lv et al. (2017)
miR-150	P2X7	Alleviate degeneration	Downregulate P2X7 → Inhibit cation influx (Na^+^, Ca^2+^, K^+^) and glial cell activation → Reduce downstream inflammatory response → Suppress NF-κB signaling and apoptosis	Zhang et al. (2019)
miR-27b-3p	Peroxisome proliferator-activated receptor gamma (PPARG)	Promote degeneration	Downregulate PPARG → Relieve PPARG-mediated suppression of NF-κB → Enhance NF-κB transcriptional activity	Guo et al. (2025)
miR-625-5p	Collagen type I alpha 1 (COL1A1)	Promote degeneration	Bind to 3’-UTR of COL1A1 → Downregulate COL1A1 → Compromise intervertebral disc structural integrity → Promote inflammatory factor-induced positive feedback loop → Activate NF-κB signaling	Shen et al. (2019)
miR-640	Low-density lipoprotein receptor-related protein 1 (LRP1)	Promote degeneration	Downregulate LRP1 → Relieve LRP1-mediated suppression of NF-κB → Activate NF-κB signaling	Dong et al. (2019)
lncRNAs				
H19	miR-139-3p	Promote degeneration	Sponge miR-139-3p → Relieve miR-139-3p-mediated inhibition of CXCR4 → Upregulate CXCR4 → Activate NF-κB signaling	Sun Z. et al. (2021)
TUG1	miR-26a	Promote degeneration	Bind to and downregulate miR-26a → Relieve miR-26a-mediated suppression of HMGB1 → Upregulate HMGB1 → HMGB1 promotes the phosphorylation and nuclear translocation of p65 → Activate NF-κB signaling	Tang et al. (2020)
circRNAs				
EYA3	miR-196a-5p	Promote degeneration	Sponge miR-196a-5p → Relieve miR-196a-5p-mediated inhibition of EBF1 → Upregulate EBF1 → EBF1 promotes phosphorylation of IκBα → Induce IκBα degradation → Release p65/p50 dimer for nuclear translocation → Activate NF-κB signaling	Wang et al. (2024)

#### 5.4.1 MicroRNAs

Certain microRNAs regulate the NF-κB pathway either positively or negatively—miR-150 and miR-217 inhibit, while miR-625-5p and miR-640 activate—yet all hold promise as IVDD therapeutic targets.

MiR-141 and miR-217 both target SIRT1 but exert opposite effects. MiR-141 downregulates SIRT1, promoting IVDD (Ji et al., 2018); conversely, miR-217, regulated by lncRNA MALAT1 carried by platelet-rich plasma-derived extracellular vesicles, upregulates SIRT1 to block NF-κB and restore ECM balance (Tao et al., 2024). MiR-146a inhibits the NF-κB pathway by downregulating TRAF6, reducing pro-inflammatory factors in NPCs (Lv et al., 2017). MiR-150 targets P2X7, an ATP-gated ion channel whose activation induces Na^+^/Ca^2+^/K^+^ influx, triggers inflammatory responses, and activates NF-κB. In IVDD, miR-150 is downregulated, leading to P2X7 elevation and subsequent NF-κB-driven inflammation and NPC apoptosis; miR-150 overexpression reverses these effects (Zhang et al., 2019).

MiR-27b-3p, miR-625-5p, and miR-640 activate NF-κB and worsen IVDD, making their inhibitors potential therapeutics. MiR-27b-3p directly targets PPARG, a negative regulator of NF-κB, thereby relieving inhibition and activating NF-κB (Guo et al., 2025). Pro-inflammatory cytokines upregulate miR-625-5p, which binds the 3′-UTR of COL1A1, reducing COL1A1 expression and compromising disc structural stability (Shen et al., 2019). Similarly, inflammatory factors elevate miR-640, which downregulates LRP1 (an indirect NF-κB inhibitor) in NPCs, activating NF-κB; additionally, miR-640 targets WNT signaling to promote NPC degeneration (Dong et al., 2019). These effects collectively accelerate IVDD progression.

#### 5.4.2 LncRNAs and circRNAs

LncRNAs and circRNAs do not directly modulate the NF-κB signaling pathway. Instead, they primarily function as sponges, interacting with and regulating downstream miRNAs to influence pathophysiological processes.

LncRNA H19 plays a crucial role in embryonic development and the regulation of growth. Previous studies have identified miR-139-3p as a target of lncRNA H19. In degenerated intervertebral discs of rats, the expression level of lncRNA H19 is elevated, and its down-regulation inhibits both autophagy and apoptosis of NPCs. Experiments conducted by Sun Z. et al. (2021) demonstrated that H19 sponges miR-139-3p to inhibit C-X-C chemokine receptor 4 (CXCR4) expression, thereby activating the NF-κB pathway, inducing autophagy and apoptosis in rat NPCs, and exacerbating IVDD.

LncRNA TUG1, similar to lncRNA H19, directly binds to miR-26a, thereby downregulating its expression in NPCs. High mobility group box 1 (HMGB1) is a target gene of miR-26a, and a negative correlation exists between miR-26a and HMGB1. Experiments conducted by Tang et al. (2020) demonstrated that HMGB1 expression in human degenerative intervertebral disc tissue is significantly higher than in healthy discs. It promotes the phosphorylation and nuclear translocation of p65 in the NF-κB p65/p50 heterodimer, thereby activating NF-κB and advancing the progression of IVDD.

CircRNAs possess a stable circular structure and exhibit a wide range of biological functions. As miRNA sponges, circRNAs specifically bind to miRNAs, thereby regulating various pathophysiological processes. For example, circEYA3 exacerbates IVDD through the miR-196a-5p/early B-cell factor 1 (EBF1) axis and the NF-κB signaling pathway. Studies show that circEYA3 functions as a sponge for miR-196a-5p, thereby indirectly modulating EBF1 expression in NPCs (Wang et al., 2024). Furthermore, EBF1 facilitates the phosphorylation of IκBα, subsequently activating the NF-κB signaling pathway, therefore exacerbating IVDD.

### 5.5 Hormones and vitamin D

Several hormones regulate NF-κB activity in IVDD, with prolactin exhibiting bidirectional effects, while estrogen, melatonin, and vitamin D predominantly exert protective actions.

Prolactin (PRL) is a pituitary peptide hormone with pleiotropic physiological roles, encompassing the regulation of reproduction, lactation, growth, and metabolism, as well as the promotion of cell proliferation, inhibition of apoptosis, and enhancement of cell survival. However, PRL exerts bidirectional regulation on the NF-κB pathway, and this duality appears to depend on species differences, cell type, or the experimental context. Wu et al. (2018) demonstrated that in a rat model of IVDD, PRL downregulated TNF-α and IL-1β expression and significantly reduced the levels of p65 and IKKα in the NF-κB signaling pathway. In contrast, Olavarría et al. (2010) reported that PRL stimulated the production of TNF-α and IL-1β and promoted NF-κB activation via Janus kinase/signal transducer and activator of transcription (Jak/Stat) signaling in leukocytes of the teleost fish. These contrasting regulatory effects of PRL on NF-κB signaling reflect fundamental differences among the experimental systems, including species divergence between mammals and teleosts, cell-type specificity between NPCs and leukocytes, and distinct physiological contexts represented by a pathological IVDD model versus basal immune stimulation. These opposing findings are not mutually exclusive; rather, they collectively suggest that PRL exerts bidirectional control over the NF-κB pathway: it may mediate anti-inflammatory effects in certain tissues or under specific conditions, while promoting inflammatory responses in other settings. Future studies are needed to delineate the specific conditions that govern the direction of PRL’s regulatory action on NF-κB signaling.

Estrogen, melatonin, and vitamin D also modulate NF-κB to protect against IVDD. Estrogen (E2) acts via ERβ: it reduces ROS to limit oxidative stress-driven NF-κB activation, suppresses apoptosis by downregulating caspase-3 and IKKα/p65 while upregulating IκBα, and inhibits MMPs through genomic effects (Wang et al., 2021; Zhang et al., 2022; Mishra et al., 2023). Melatonin can inhibit the NF-κB signaling pathway by suppressing the phosphorylation and degradation of IκB*α*, thereby reducing the nuclear translocation of p65. In addition, in degenerated NP tissues, LC3B and autophagosomes are decreased while p62 is increased, indicating impaired autophagy. Melatonin can also delay IVDD by inducing autophagy. Vitamin D blocks NF-κB by lowering p65 and IKKα, elevating SOD-1/−2, decreasing IL-1β/TNF-α, reducing caspase-3/−8, and increasing Bcl-2, thereby counteracting inflammation, oxidative stress, and apoptosis (Huang et al., 2019; Liu et al., 2022). Collectively, these hormones offer multifaceted therapeutic targets for IVDD management.

### 5.6 Natural compounds

Natural compounds refer to substances that occur naturally and have not been synthetically produced. Natural medicines derived from animals, plants, and microorganisms offer distinct advantages over synthetic drugs, as they can target multiple pathways simultaneously, thereby providing a more comprehensive therapeutic effect. Additionally, natural medicines are associated with fewer adverse reactions and exhibit fewer side effects on the human body (Chaughule and Barve, 2023; Salm et al., 2023). In recent years, natural compounds have been extensively investigated in the context of IVDD. The majority of natural compounds targeting the NF-κB pathway to treat IVDD are derived from plants. Based on the classification of organic compounds, these natural drugs can primarily be categorized into flavonoids, alkaloids, terpenes, and saponins (Rong et al., 2022). However, some natural compounds fall outside these categories. For example, amygdalin is classified as a glycoside, curcumin as a phenol, phillyrin as a lignan, and chlorogenic acid as a phenylpropanoid (Zeng et al., 2023; Ma et al., 2015; Ge et al., 2021; Hamdalla et al., 2022). These compounds can also treat IVDD by preventing NF-κB activation (Table 2).

**Table 2:** Therapeutic effects of natural compounds on IVDD.

Natural compounds	Target	Main effects	References
Flavonoids			
Mulberroside A	Ppar-γ/ p65, p50	Activate Ppar-γPpar-γ binds to p65, p50 dimers	Xu et al. (2024)
Hesperidin	Nrf2/ NF-κB	Activate Nrf2 to reduce oxidative stress inhibit the NF-κb signaling pathway to attenuate the inflammatory response	Zhu et al. (2023)
Myricetin	Nrf2	Activate Nrf2 to reduce oxidative stress	Mao and Fan (2024)
Quercetin	Nrf2	Activate Nrf2 to reduce oxidative stress	Shao et al. (2021)
Galangin	Nrf2	Activate Nrf2 to reduce oxidative stress	Chen L. et al. (2025)
Mangiferin	p65/ROS	Inhibit p65 nuclear translocation reduce ROS in NPCs	Yu et al. (2021)
Homoplantaginin	TAK1/ p65	Bind to TAK1 inhibit P65 phosphorylation	Li B. et al. (2024)
Velutin	p65/MAPK	Inhibit p65 nuclear translocation inhibit the MAPK signaling pathway	Zhang et al. (2025)
Aucubin	p65	Inhibit P65 phosphorylation	Zou et al. (2023)
Alkaloids			
Stachydrine	PI3K/Akt/ NF-κB	Tightly integrate with PI3K inhibit the PI3K/AKT pathway inhibit the NF-κB pathway	Shao et al. (2020)
Lycorine	IκBα	Inhibit phosphorylation of IκBα	Wang G. et al. (2018)
Sinomenine	Nrf2/ NF-κB	Activate Nrf2 to reduce oxidative stress inhibit the NF-κB pathway	Lu et al. (2023)
Magnoflorine	HMGB1/ MyD88/p65	Inhibit the production of HMGB1 by NPCs reduce the expression levels of MyD88 and p65	Zhao F. et al. (2021)
Terpenes			
Maslinic acid	PI3K/Akt/ NF-κB	Tightly integrate with PI3K inhibit the PI3K/AKT pathway inhibit the NF-κB pathway	Que et al. (2024)
Dihydroartemisinin	PI3K/Akt/ NF-κB	Tightly integrate with PI3K inhibit the PI3K/AKT pathway inhibit the NF-κB pathway	Liao et al. (2022)
Limonin	MAPK/ P65	Inhibit the MAPK signaling pathway inhibit P65 phosphorylation	Gong et al. (2023)
Saponins			
Ginsenoside Rg1	P65/NPCs	Inhibit P65 phosphorylation promote the proliferation of NPCs inhibit apoptosis of NPCs	Yu et al. (2024)
Astragaloside IV	P65/NPCs	Inhibit P65 phosphorylation promote the proliferation of NPCs inhibit apoptosis of NPCs	Tian et al. (2022)

#### 5.6.1 Flavonoids

Mulberroside A is a natural compound extracted from mulberries (Xu et al., 2024). It inhibits the NF-κB pathway primarily by activating PPAR-*γ*. Mulberroside A acts as a PPAR-γ agonist, binding to p65 and p50 dimers, therefore suppressing the NF-κB signaling cascade and aiding in the treatment of IVDD. Hesperidin, a flavonoid abundant in citrus fruits, has also been shown to alleviate IVDD (Zhu et al., 2023). First, hesperidin mitigates the inflammatory response by obstructing the NF-κB signaling pathway. Additionally, hesperidin activates Nrf2, which reduces oxidative stress-induced damage to NPCs. In NPCs treated with hesperidin, the expression of NOX2 and NOX4 is reduced, while the levels of SOD-1 and HO-1 are increased. These findings demonstrate that hesperidin alleviates IVDD through multiple mechanisms. Similarly, myricetin, galangin and the anti-aging agent quercetin inhibit the nuclear translocation of p65 by activating Nrf2, thereby blocking the NF-κB signaling pathway (Mao and Fan, 2024; Shao et al., 2021; Chen L. et al., 2025). Mangiferin and hesperidin exhibit similar effects in inhibiting oxidative stress and the inflammatory response associated with IVDD. Experiments by Yu et al. (2021) demonstrated that mangiferin can prevent the nuclear translocation of p65 induced by pro-inflammatory factors such as TNF-α, while also reducing ROS levels in NPCs. Traditional Chinese herbs also play a role in the treatment of IVDD. Homoplantaginin, derived from lychee grass, may directly interact with TAK1 in the NF-κB pathway, therefore reducing the phosphorylation of p65 (Li B. et al., 2024). Engeletin, found in mugwort, and Velutin inhibit both the NF-κB and MAPK pathways (Zhang et al., 2025; Li et al., 2022). Aucubin is widely distributed in nature, found in plants such as plantain, figwort, and rehmannia, as well as other Chinese herbal medicines. Studies have shown that p65 expression is significantly reduced in cells treated with aucubin, both *in vivo* and *in vitro* (Zou et al., 2023). These findings show that the NF-κB pathway is likewise suppressed by aucubin.

#### 5.6.2 Alkaloids

The primary alkaloid compounds that inhibit NF-κB pathway activation include stachydrine, lycorine, sinomenine, and magnoflorine. Stachydrine is the major component of the Chinese herb Motherwort. It treats IVDD through the crosstalk between the PI3K/Akt and NF-κB signaling pathways. The PI3K/Akt pathway acts upstream of the NF-κB pathway. Stachydrine helps cure IVDD by blocking the PI3K/Akt signaling pathway, which lowers the nuclear translocation of p65 (Shao et al., 2020). Lycorine’s influence on the NF-κB signaling pathway is largely understood. By preventing IκBα from being phosphorylated, lycorine suppresses the NF-κB pathway in endplate chondrocytes (Wang G. et al., 2018). However, it does not significantly affect the MAPK pathway, and its therapeutic effect on IVDD appears relatively straightforward. Sinomenine exhibits strong therapeutic effects on oxidative stress and the inflammatory response during IVDD progression. Experiments by Lu et al. (2023) have shown that sinomenine reduces inflammation by blocking the NF-κB pathway and alleviates oxidative stress through the Keap1/Nrf2 signaling pathway. When NPCs are damaged by oxidative stress, Keap1 fails to ubiquitinate Nrf2, leading to Nrf2 phosphorylation and its subsequent translocation to the nucleus, where it induces the expression of antioxidant enzymes. The approach of magnoflorine in treating IVDD is relatively unique. Experiments by Zhao F. et al. (2021) demonstrated that NPCs and M1-activated macrophages form a positive feedback loop. Degenerated NPCs produce HMGB1 in response to inflammatory factors, which then acts on macrophages to polarize them toward the M1 phenotype. The generation of inflammatory factors that worsen NPC damage is caused by the activation of the HMGB1-MyD88-NF-κB pathway in M1 macrophages. However, magnoflorine disrupts this loop by inhibiting HMGB1 production in NPCs and reducing the expression of MyD88 and p65.

#### 5.6.3 Terpenes and saponins

Maslinic acid (MA) and dihydroartemisinin act in a manner similar to stachydrine. All of them regulate the NF-κB pathway by inhibiting the PI3K/AKT pathway, thereby mitigating IVDD (Que et al., 2024; Liao et al., 2022). Both MA and dihydroartemisinin tightly bind to PI3K, inhibiting the PI3K/AKT pathway. Since the PI3K/Akt pathway acts upstream of the NF-κB pathway, its inhibition leads to the suppression of NF-κB signaling and the treatment of IVDD. Limonin is commonly found in citrus fruits. Gong et al. (2023) have demonstrated experimentally that limonin alleviates IVDD via blocking the MAPK and NF-κB pathways. Regarding the MAPK pathway, limonin inhibits the expression of phosphorylated JNK, ERK, and P38 proteins. In the NF-κB pathway, it inhibits P-p65 expression. Ginsenoside Rg1 and astragaloside IV are saponin compounds extracted from ginseng and astragalus, two traditional Chinese herbs. Previous studies have shown that the p-p65/p65 ratio in NPCs significantly decreased following treatment with ginsenoside Rg1 and astragaloside IV (Yu et al., 2024; Tian et al., 2022). Ginsenoside Rg1 also inhibits NPC apoptosis and promotes their proliferation, which helps restore the balance of ECM synthesis and metabolism (Jin W. et al., 2026; Yu L. et al., 2020). These natural compounds derived from Chinese herbal medicine offer potential new targets and treatments for IVDD.

### 5.7 Novel biomaterials

#### 5.7.1 Nanomaterials

Tu et al. (2023) designed a nanofiber scaffold capable of sustaining the release of basic fibroblast growth factor (bFGF), which promotes the regeneration of annulus fibrosus cells (AFCs). In addition, this scaffold incorporates type I collagen, fostering an optimal microenvironment that supports AFCs’ adhesion and proliferation. Yang W. et al. (2024) developed a nanoparticle sponge (MnO2@TMNP) with antioxidative and anti-inflammatory properties for treating degenerated intervertebral discs. This innovative design encapsulates MnO2 nanoparticles within macrophage membranes. The macrophage membranes enable targeted delivery to macrophages while functioning as a molecular sponge that absorbs pro-inflammatory factors and NGF, a critical contributor to discogenic pain in IVDD. Similarly, Li K. et al. (2024) developed TMNP@SR, which utilizes TrkA-rich macrophage membranes as its outer layer, resembling MnO2@TMNP (Zhou et al., 2024). However, TMNP@SR incorporates mesoporous silica loaded with rapamycin (SR) at its core, promoting macrophage polarization from the M1 to the M2 phenotype. Additionally, Zhou et al. (2024) developed a multifunctional nanoparticle, PG@Cu-FP, to address IVDD. PG@Cu-FP includes pentapeptides that specifically target mitochondria, enabling the precise elimination of excess ROS. Moreover, it suppresses the production of proteins linked to apoptosis, including Bax and Caspase 9, so it can stop NPCs from dying and preserve the homeostasis of the extracellular matrix. Finally, PG@Cu-FP suppresses NLRP3 inflammasome activation, thereby reducing pro-inflammatory factors linked to IVDD.

#### 5.7.2 Hydrogel materials

IVDD leads to structural destruction in the NP, AF, and CEPs. Owing to their excellent tunability and biocompatibility, hydrogels can be tailored to repair individual structures of the degenerated intervertebral disc. The following examples illustrate how different hydrogels are specifically engineered to target the NP, AF, and CEPs damaged in IVDD.

For the NP, GM@CS-BP reduces oxidative stress and inhibits p65 phosphorylation to lower pro-inflammatory factors (Li Z. et al., 2023); pH-responsive hydrogels co-loaded with IL-37 and itaconate suppress NF-κB to reduce SASP and regulate macrophage polarization (Li et al., 2025); and gelatin methacrylate (GelMA) hydrogels with 10-hydroxy-2-decenoic acid (10-HDA) inhibit NF-κB while targeting ferroptosis to reduce NPC apoptosis (Zhang and Yang, 2025). For the AF, an innovative hydrogel releasing TGF-β1, combined with collagen mimetic peptide (CMP) modifications, not only chemoattracts AFCs and promotes their proliferation, but also modulates the local microenvironment to enhance AFC recruitment and indirectly support their regenerative capacity. Concurrently, it inhibits p65 phosphorylation to suppress NF-κB and reduce inflammation (Wei et al., 2024). For the CEPs, Zhan et al. (2025) developed a hydrogel named CAP-sEXOs@Gel. It delivers CEPC-targeted exosomes that inhibit oxidative stress, restrict iron influx, and suppress cyclic GMP-AMP synthase – stimulator of interferon genes (cGAS-STING) activation, thereby preventing CEPC degeneration and restoring ECM components.

In summary, these hydrogels are individually tailored for the NP, AF, or CEPs, yet they share a common goal of restoring the structural integrity of the disc. By separately targeting each damaged compartment, they together provide a comprehensive framework for IVDD repair.

#### 5.7.3 Microneedles (MNs)

Nanomaterials and hydrogel-based systems are widely utilized as drug carriers to deliver therapeutic agents to target cells, mitigating IVDD. However, controlling the final drug concentration delivered to the intervertebral disc remains challenging. To address this, Zhang et al. (2024) designed a microneedle system capable of modulating exosome release and TREM1 levels based on mechanical movement intensity. Given that excessive mechanical loading is a key contributor to IVDD, the required drug dosage should correlate positively with mechanical activity levels. The microneedle incorporates a triboelectric nanogenerator (TENG), which converts mechanical energy from movement into electrical energy. The resulting electric field modulates the state of polypyrrole (PPy) within the microneedle. Due to energy conservation during this process, the amount of exosomes released is directly proportional to the intensity of mechanical movement.

Microneedle-delivered exosomes can be engineered to carry the TRAM1 protein. TRAM1 binds to TREX1 within cells, anchoring it to the endoplasmic reticulum and preventing its nuclear translocation. In IVDD, the TRAM1-TREX1 complex undergoes significant degradation, allowing TREX1 to translocate into the nucleus, where it damages genomic DNA. Once in the nucleus, TREX1 activates the cGAS-STING pathway, elevating pro-inflammatory factor levels in the intervertebral disc. These microneedles deliver TRAM1 protein into damaged NPCs, delaying cellular dysfunction and offering a therapeutic approach to IVDD.

## 6 Targeting NF-κB in IVDD: challenges and future perspectives

Current therapeutic strategies targeting the NF-κB pathway in IVDD can be broadly categorized into four classes: small-molecule agents (including NSAIDs, certain hormones, and natural compounds), macromolecular biologics (proteins, peptides, and growth factors), nucleic acid therapeutics (non-coding RNAs), and advanced biomaterial-based delivery systems. These strategies each have distinct characteristics in terms of drug delivery, clinical application, and safety, yet they also face respective challenges and exhibit differentiated developmental trajectories.

### 6.1 Comparison of diverse therapeutic strategies

Small-molecule drugs such as NSAIDs are widely used in clinical practice, primarily administered orally or by injection to alleviate lower back pain. However, they cannot fundamentally reverse or halt the progression of intervertebral disc degeneration, and long-term use carries risks of gastrointestinal and cardiovascular adverse effects. Estrogen, melatonin, and vitamins can inhibit NF-κB through endogenous regulatory mechanisms and exhibit good biocompatibility. Nevertheless, these agents are mostly in the preclinical stage, with their clinical efficacy not fully evaluated, and they may also cause hormonal imbalances. Natural compounds have become a research hotspot in IVDD in recent years. Most of them are derived from active ingredients extracted from natural plants and demonstrate good safety profiles. Additionally, it also has multi-target activity, which can regulate pathways such as NF-κB, Nrf2, and MAPK, exerting synergistic therapeutic effects on multiple aspects of IVDD. However, most drugs are still at the animal experiment stage and lack a comprehensive clinical treatment plan.

Macromolecular biologics such as proteins and peptides have a clear targeting specificity for NF-κB, sparing the side effects associated with broad-spectrum inhibitors. Additionally, these substances are mostly endogenous molecules with good biocompatibility. For growth factors, platelet-rich plasma (PRP) injection therapy carries multiple autologous growth factors that alter the NF-κB signaling pathway to treat IVDD. However, this approach faces the challenge that growth factors have a short half-life *in vivo* and are prone to rapid degradation (Huang et al., 2023). The hydrogel-based delivery system can achieve slow and sustained release of growth factors, resulting in long-term therapeutic effects. Chen G. et al. (2025) developed a bio-engineered thermo-sensitive injectable hydrogel loaded with GDF-5, which enhanced nucleus pulposus cell activity and inhibited inflammation *in vitro* and effectively promoted nucleus pulposus regeneration and intervertebral disc functional recovery in a rat model. However, due to issues such as the long-term stability and biocompatibility of its hydrogel, it is still in the preclinical stage.

Nucleic acid therapy can precisely regulate the NF-κB pathway at the post-transcriptional level, offering sequence specificity that traditional drugs cannot match. However, naked nucleic acids are highly susceptible to degradation by nucleases within the intervertebral disc, necessitating appropriate carriers to transport them to the site of action. Advanced biomaterials precisely compensate for this delivery deficiency: they not only protect nucleic acids from degradation, enable sustained release and targeted delivery, but also actively scavenge ROS, reprogram macrophage polarization, and modulate the degenerative microenvironment (Liu et al., 2026). However, biomaterials lack precise regulatory capabilities and face limitations such as high batch-to-batch variability and unknown long-term biocompatibility. Currently, both strategies are in the preclinical stage for IVDD treatment.

In summary, small-molecule drugs have a broad range of action and can alleviate IVDD through multiple pathways, but they lack specificity and may cause side effects. Macromolecular biologics and nucleic acid therapies have good targeting ability and biocompatibility, but they face challenges of insufficient stability within the intervertebral disc and delivery issues. Advanced biomaterials themselves also have the function of alleviating IVDD, but they cannot be precisely regulated and are mostly used as carriers for therapeutic drugs.

### 6.2 Key challenges in targeted NF-κB therapy for IVDD

Targeting the NF-κB pathway plays a key role in the treatment of IVDD, but most investigational drugs remain in the preclinical stage, and there are difficulties in the clinical translation of these drugs.

First, the unique physiological structure of the intervertebral disc makes it difficult to deliver therapeutic agents to its interior. The intervertebral disc is the largest avascular tissue in the human body, and its nutritional supply relies entirely on diffusion from the CEP and AP. As IVDD progresses, calcification and sclerosis of the CEP further exacerbate this barrier, making it impossible for oral or subcutaneous administration to achieve an effective concentration within the intervertebral disc. Direct intradiscal injection cannot facilitate slow and uniform diffusion of drugs, causing them to accumulate near the injection site without effective distribution. Moreover, due to the influence of the acidic, hypoxic, and ROS microenvironment within the intervertebral disc, injected drugs such as nucleic acids, proteins, and peptides are rapidly cleared, hindering the assurance of long-term therapeutic efficacy. Regarding the delivery of therapeutic agents, current approaches often utilize hydrogels, exosomes, and other materials to achieve slow and sustained drug release, while also improving the controllability of drug release in response to the microenvironment of the degenerative intervertebral disc. However, the complex manufacturing processes, biocompatibility, and clinical safety of these materials limit their development. Innovation in delivery systems is as crucial as the development of NF-κB pathway inhibitors, representing a core bottleneck in molecular therapy for IVDD.

Additionally, we must consider the specificity and safety of drugs targeting NF-κB. NF-κB is a core factor regulating inflammatory responses and immune defense, and its systemic over-inhibition will disrupt physiological immune homeostasis, increase susceptibility to infections, and may induce autoimmune disorders (Sun Y. et al., 2024). Therefore, therapeutic drugs need to be highly targeted to the degenerated intervertebral disc, achieving high concentrations in the IVDD region while maintaining low systemic exposure, so as to ensure therapeutic efficacy while reducing side effects. Finally, we need to clarify the therapeutic window for drugs targeting NF-κB in the treatment of lumbar IVDD. The early stage of IVDD is primarily characterized by reversible inflammatory responses and imbalance of the disc microenvironment, while the late stage involves massive loss of nucleus pulposus cells and structural deterioration of the extracellular matrix. It is necessary to specify that different therapeutic windows correspond to different drugs and treatment regimens. Moreover, most of the aforementioned therapeutic drugs are still at the preclinical stage, and animal experiments are often modeled using acute spinal cord injury, where the dimensions and biomechanical loads of the intervertebral discs differ from those of the human lumbar spine. In addition, human IVDD is a chronic and slow process, which also presents significant differences. Further research is needed to validate the efficacy and safety of targeting NF-κB for IVDD treatment in humans.

### 6.3 Future research directions

As research continues to deepen, the emergence of various novel drugs and materials has provided new ideas for targeted NF-κB therapy in IVDD.

First, responsive materials should be designed according to the degree of intervertebral disc degeneration. In view of the special microenvironment of degenerated discs, the drug should be released only upon activation in the degenerative microenvironment while remaining inert in healthy tissues, thereby balancing local efficacy and safety. For example, the microneedle system mentioned above can modulate drug release based on the magnitude of mechanical stress, thus achieving precise treatment of IVDD. Second, multi-omics technologies have advanced the development of therapeutic strategies for IVDD. By integrating transcriptomic, proteomic, metabolomic and epigenomic data, it is possible to precisely identify degenerative subtypes and molecular stages and to screen reliable biomarkers for patient stratification and optimal intervention timing. This multi-omics approach helps us understand the interactions among genes, proteins, and metabolites, thereby providing a basis for personalized therapy. Third, the non-canonical NF-κB pathway has unique functions in immune regulation and inflammation, and its aberrant activation is closely associated with diseases such as rheumatoid arthritis. In the context of IVDD, this pathway may exert effects through regulating nucleus pulposus cell survival, inflammatory responses, and matrix metabolism. Small-molecule inhibitors targeting NIK or IKKα have shown therapeutic potential in other inflammatory models, suggesting that they may offer new intervention targets for IVDD (Sun, 2017). Finally, to promote the clinical application of carrier materials, we need to compare their stability under the disc microenvironment and their drug release and distribution characteristics. This will help establish standardized evaluation systems in the future, clarify the optimal application scenarios for each carrier, and provide direct evidence for carrier selection in clinical translation.

## 7 Conclusion

This review focuses on therapeutic targets that inhibit IVDD progression through the NF-κB pathway. In IVDD, decreased BCL-2 promotes NPC apoptosis and reduces ECM synthesis, while elevated MMPs accelerate ECM degradation, depleting collagen II, aggrecan, and NP water content. Multiple predisposing factors create a pro-inflammatory milieu that activates NF-κB in NPCs, establishing a positive feedback loop that amplifies inflammation. The normally avascular, immune-privileged disc loses this protection during IVDD, allowing immune cell infiltration that further drives NF-κB-mediated cascades. Given this central role, diverse therapeutic agents have been explored. They can directly block the phosphorylation/nuclear translocation of IKKβ/p65, or indirectly modulate through crosstalk with signaling pathways such as MAPK (Zhu et al., 2024; Luo et al., 2022; Bang et al., 2021). Among these, plant-derived natural compounds have gained attention for their broad efficacy and low toxicity (Samuel et al., 2021). Advanced biomaterials themselves can also alleviate IVDD and serve as carriers for sustained, targeted delivery, thereby enhancing therapeutic outcomes.

Targeting NF-κB shows promise for IVDD treatment. However, current strategies face substantial limitations. Most targeted agents remain experimental. Their clinical efficacy and side effects have not been validated. The non-canonical activation pathway has not been specifically investigated in IVDD. The almost avascular disc microenvironment poses a major challenge for precise drug delivery. This greatly compromises the effectiveness of NF-κB-targeted interventions. Moreover, IVDD arises from the interplay of various signaling pathways. Therefore, inhibiting NF-κB alone must account for crosstalk with other pathways. Future research should develop smart delivery systems responsive to disc microenvironmental cues. The suitability of carriers under hypoxic and avascular conditions needs systematic evaluation. Multi-omics can help identify biomarkers for personalized therapy. Elucidating non-canonical pathway nodes such as NIK/IKKα will accelerate clinical translation. Despite the long road to clinical application, the NF-κB pathway undoubtedly offers a promising breakthrough direction for IVDD management.
